# Characterizing and Modeling Smoking Behavior Using Automatic Smoking Event Detection and Mobile Surveys in Naturalistic Environments: Observational Study

**DOI:** 10.2196/28159

**Published:** 2022-02-18

**Authors:** DongHui Zhai, Ruud van Stiphout, Giuseppina Schiavone, Walter De Raedt, Chris Van Hoof

**Affiliations:** 1 Department of Electrical Engineering Katholieke Universiteit Leuven Leuven Belgium; 2 imec Leuven Belgium; 3 imec at OnePlanet Research Center Wageningen Netherlands

**Keywords:** smoking behavior modeling, ambulatory study, wearable sensors, temporal patterns of smoking, Poisson mixed-effects model, mobile phone

## Abstract

**Background:**

There are 1.1 billion smokers worldwide, and each year, more than 8 million die prematurely because of cigarette smoking. More than half of current smokers make a serious quit every year. Nonetheless, 90% of unaided quitters relapse within the first 4 weeks of quitting due to the lack of limited access to cost-effective and efficient smoking cessation tools in their daily lives.

**Objective:**

This study aims to enable quantified monitoring of ambulatory smoking behavior 24/7 in real life by using continuous and automatic measurement techniques and identifying and characterizing smoking patterns using longitudinal contextual signals. This work also intends to provide guidance and insights into the design and deployment of technology-enabled smoking cessation applications in naturalistic environments.

**Methods:**

A 4-week observational study consisting of 46 smokers was conducted in both working and personal life environments. An electric lighter and a smartphone with an experimental app were used to track smoking events and acquire concurrent contextual signals. In addition, the app was used to prompt smoking-contingent ecological momentary assessment (EMA) surveys. The smoking rate was assessed based on the timestamps of smoking and linked statistically to demographics, time, and EMA surveys. A Poisson mixed-effects model to predict smoking rate in 1-hour windows was developed to assess the contribution of each predictor.

**Results:**

In total, 8639 cigarettes and 1839 EMA surveys were tracked over 902 participant days. Most smokers were found to have an inaccurate and often biased estimate of their daily smoking rate compared with the measured smoking rate. Specifically, 74% (34/46) of the smokers made more than one (mean 4.7, SD 4.2 cigarettes per day) wrong estimate, and 70% (32/46) of the smokers overestimated it. On the basis of the timestamp of the tracked smoking events, smoking rates were visualized at different hours and were found to gradually increase and peak at 6 PM in the day. In addition, a 1- to 2-hour shift in smoking patterns was observed between weekdays and weekends. When moderate and heavy smokers were compared with light smokers, their ages (*P*<.05), Fagerström Test of Nicotine Dependence (*P*=.01), craving level (*P*<.001), enjoyment of cigarettes (*P*<.001), difficulty resisting smoking (*P*<.001), emotional valence (*P*<.001), and arousal (*P*<.001) were all found to be significantly different. In the Poisson mixed-effects model, the number of cigarettes smoked in a 1-hour time window was highly dependent on the smoking status of an individual (*P*<.001) and was explained by hour (*P*=.02) and age (*P*=.005).

**Conclusions:**

This study reported the high potential and challenges of using an electronic lighter for smoking annotation and smoking-triggered EMAs in an ambulant environment. These results also validate the techniques for smoking behavior monitoring and pave the way for the design and deployment of technology-enabled smoking cessation applications.

**International Registered Report Identifier (IRRID):**

RR2-10.1136/bmjopen-2018-028284

## Introduction

### Background

After more than 100 years of popularity, cigarette smoking remains the single largest cause of preventable disease and death even in the 21st century [1,2]. Globally, there are 1.1 billion current smokers, and every year more than 8 million die prematurely because of smoking. In addition, smoking induces many other health and economic costs on the society [2]. Although more than half of current smokers make a serious attempt to quit each year in the United States [3], 90% of unaided quitters relapse within the first 4 weeks due to the lack of limited access to cost-effective and efficacious smoking cessation tools [4], and only around 2% can quit for good. If no better solutions are developed to help increase the success ratio of smoking abstinence, the prevalence of smoking will decline very slowly and can be expected to remain at high levels for decades into the future [5]. Nonetheless, for designing readily accessible and effective smoking cessation applications, there are generally 2 obstacles ahead.

The first challenge is the lack of appropriate tools for smoking prevention and monitoring in daily use. Primary care plays a central role in smoking cessation, but its high-quality services are costly and often constrained by physical factors such as distance or time [6]. In fact, only 8% of the smokers go to smoking cessation clinics or physicians for counseling when they try to quit smoking [7]. In addition, smoker-initiated retrospective reports or diaries are the main methods used in previous studies on smoking research, but there are challenges with synchronizing events with digitalized measurements and recall of annotations by participants. To overcome this barrier, researchers have been designing and using many smart gadgets for smoking behavior monitoring and modeling. For example, radio frequency sensors and inertial sensors to measure respiration and arm movements have been used for smoking detection [8,9]. Acoustic sensors and breath carbon monoxide sensors were used for monitoring smoking, in combination with electric lighters and wrist-worn sensors [10,11]. Finally, the most recent use of e-cigarettes makes it easier to track and model this behavior [12].

The second challenge is how to transform theoretical models on smoking into actionable guidance tools in the dynamic context of daily use [13]. Many existing smoking cessation apps only use simplistic tools such as calculators, educational text [14], photoaging images [15], and self-trackers [16] and fall short of providing features such as smart tracking, learning, and tailored feedback, which are mostly demanded by end users. To enable adaptive smoking interventions, a prerequisite is to collect multimodal data concurrent with smoking and then use them to analyze the temporal and contextual windows associated with smoking behavior. In the literature, a few groups have reported progress in this direction. For example, Saleheen et al [9] designed a multi-sensor approach (electrocardiography, 3-axis accelerometer, and respiration sensors) and used it to collect smoking-related data from 45 smokers. Later, they conducted a study with 55 participants to test their app (MyQuitPal) designed for smokers during their initial cessation process [17,18]. However, it was only used among hospitalized smokers for 4 days and mainly to test various visualization techniques of their prototype system with no evaluation of its efficacy.

Accurate monitoring and modeling of smoking behavior in real-life settings are crucial for designing and delivering appropriate smoking cessation interventions. To fulfill this goal, mobile health technology, combining the measurement of multimodal sensors with the computation power of ubiquitous mobile phones, could enable a quantified observation of ambulatory smoking behavior 24/7 in real life. Because this technique can capture diverse information relevant to the behavior of interest, it can not only support accurate analysis and modeling of smoking behavior but also deliver customized interventions.

### Objectives

The aim of this study is to acquire a better understanding of smoking behavior by analyzing data from a longitudinal study, in which smoking events were automatically tracked and smoking-contingent context and mood states were assessed using mobile technology.

## Methods

### Study

This was an observational study of smoking behavior in a real-life setting, following the protocol reported in [19]. Smoker volunteers were recruited from the Flanders area of Belgium to participate in a 4-week experiment. Inclusion criteria were adults aged between 18 and 65 years, current smokers, office workers, and with no psychological, cardiac, or respiratory problems. An intake questionnaire and informed consent form were filled out when the participants passed the screening phase and were registered for this study. The intake questions were about personal background information such as age, gender, BMI, and the 6-item Fagerström Test of Nicotine Dependence (FTND). The FTND is a validated standardized smoking assessment that can be converted to a final score ranging from 0 to 10 and is used to indicate the nicotine dependence of smokers [20]. The FTND score measures physiological dependence (ie, tolerance and withdrawal). However, it does not capture the behavioral and psychosocial dimensions of nicotine dependence [21].

When the experiment began, the participants downloaded and installed an experimental app called ASSIST [19] on their smartphone. Next, they were given 2 wearable sensors, 1 electrical lighter, and instructions on the use of these sensors and the app. They were also informed to solely use the assigned lighter to light cigarettes when they smoke, and they were asked not to share it with other smokers. The lighter was also connected to the app on their phone via Bluetooth and was used to trigger surveys.

### Ecological Momentary Assessment Surveys

Ecological momentary assessment (EMA) surveys have been used in many experiments to study smoking behavior to capture, for example, environmental factors and affect, which are common reasons for smoking relapse [22]. EMAs aim to capture more reliable experience sampling because of their more relevant timing around the event of smoking, as reported by Serre et al [23].

In the current design of this study, participants were prompted to make annotations about their emotional state such as affect and arousal, dependence symptoms such as craving, enjoyment of cigarettes, and difficulty resisting smoking and other contexts related to smoking (social, activity, etc). These prompts were primarily triggered by the smoking events captured by the electric lighter. To prevent smokers, especially heavy smokers, from overburden, EMA surveys could only pop up at least 45 minutes apart. In addition, when the Bluetooth connection was down, the triggering fell back on a predefined randomization mechanism. In such cases, users received at most 5 randomized surveys per day.

### Statistical Analysis

The EMA score correlations were assessed using the Spearman correlation coefficient. Comparison of the smoker groups (light smokers: ≤10 cigarettes per day vs moderate to heavy smokers: >10 cigarettes per day) was performed using the Mann-Whitney *U* test for continuous variables, including EMA variables (assuming a sufficient range of discrete values). A generalized Poisson regression model from the GLMMadaptive package in R (R Foundation for Statistical Computing) was used to model smoking rates in 1-hour windows [24]. This model was selected because hourly smoking rates followed a Poisson distribution with 1 cigarette being the most common value and a rapid decline for a higher number of cigarettes smoked.

## Results

### Data Set and User Statistics

In total, 52 adult smokers volunteered to participate in the study, but, of these, 6 (12%) decided to quit the study and were excluded from the data set. Table 1 lists the characteristics of the data set. Of the 46 participants, 28 (61%) were men and 18 (39%) were women, with a mean age of 36 (SD 9.9) years. In all, 26% (12/46) of participants did not report their BMI, and the rest (34/46, 74%) had a mean BMI of 25 (SD 4.8) kg/m^2^.

Regarding FTND, no smokers were assessed with high nicotine dependence in the study; all the smokers belonged to the first 3 groups. During the experiment, 8639 cigarettes were tracked by the lighter over 902 participant days. Specifically, 67% (31/46) of participants smoked ≤10 cigarettes a day on average and were labeled as light smokers. A total of 28% (13/46) of moderate smokers consumed 10-20 cigarettes on average daily. Only 4% (2/46) of heavy smokers had smoked >20 cigarettes a day. In contrast, there were 52% (24/46) of light smokers and 44% (20/46) of moderate smokers, according to the self-reported average daily cigarette consumption.

**Figure 1:**
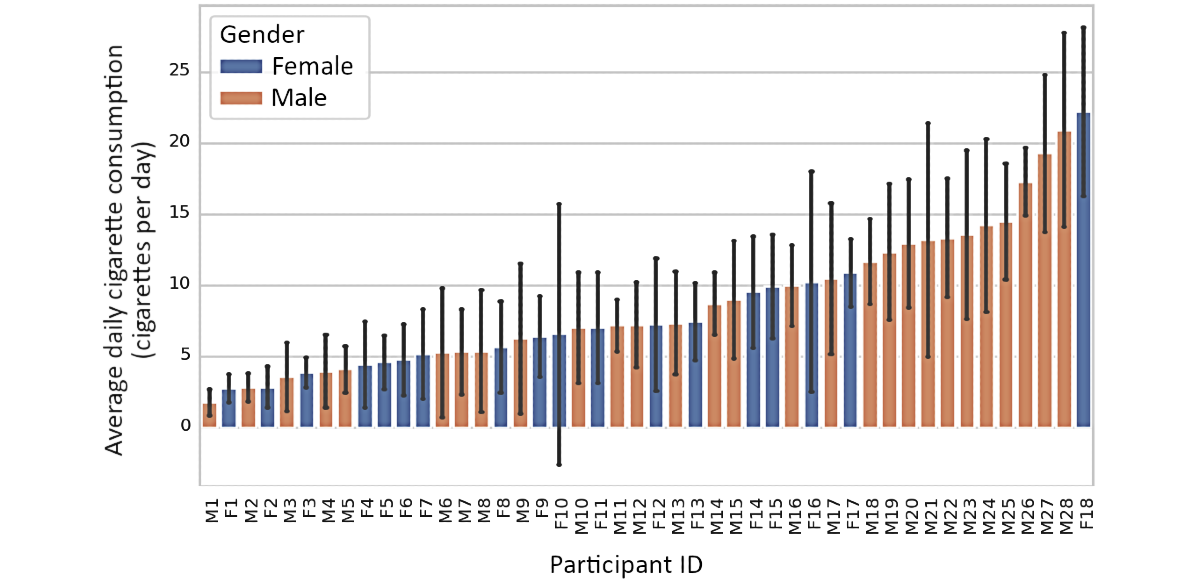
The average number of cigarettes smoked daily by each participant during the experiment. The colored bars and black lines represent the mean and SD, respectively. The participants on the x-axis are sorted by the mean in ascending order. F: female; M: male.

**Figure 2:**
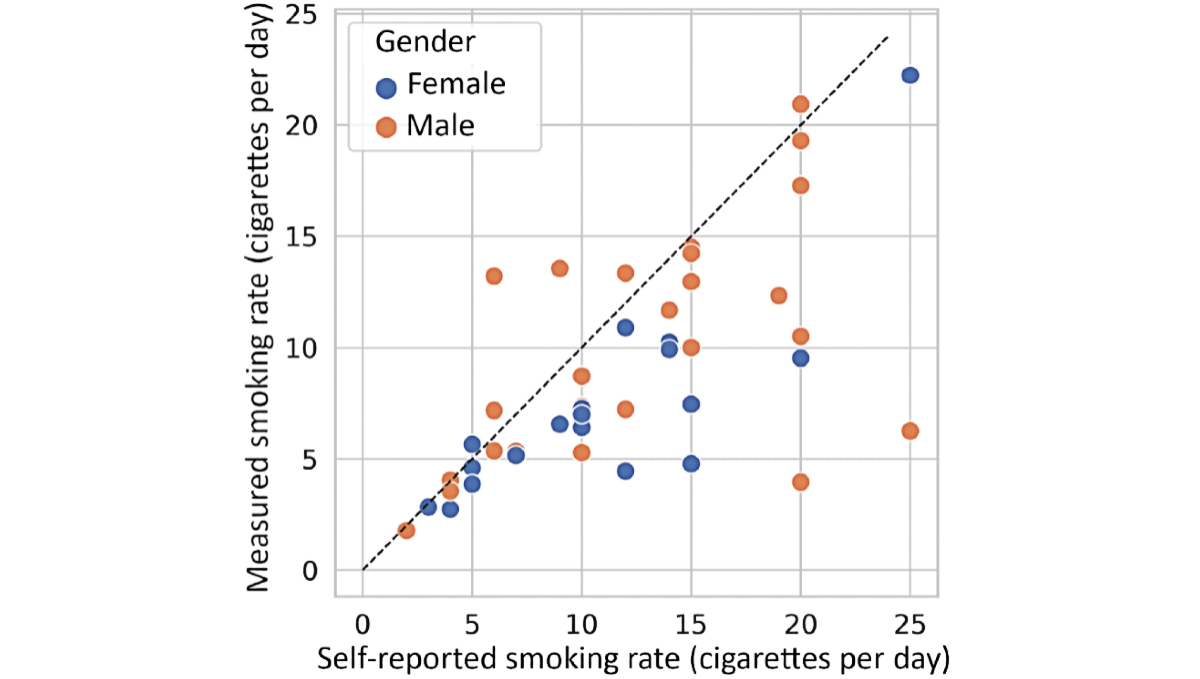
Comparison of the self-reported average number of cigarettes smoked daily with the measured consumption by the electric lighter. The dashed line is the diagonal of equal values. Gender is specified by colored markers as depicted in legend.

Figure 1 shows the average and SD of cigarettes smoked daily by smokers in this study. The participants were ranked by average daily consumption of cigarettes. We observed that most smokers had a day-to-day variation >1 cigarette per day (CPD), except for the first light smoker M1. Specifically, 72% (33/46) of the smokers had a moderate variation of 2 to 5 CPD, 20% (9/46) with a variation of 5 to 7 CPD, and 7% (3/46) with a variation >7 CPD based on the measured smoking records [11,25,26]. Figure 2 compares the self-reported number of cigarettes and objectively measured ones with the electric lighter. It shows that 74% (34/46) of the smokers made estimations that deviated more than one cigarette (mean 4.7 per day, SD 4.2 per day), and 70% (32/46) overestimated it compared with the lighter measurements.

**Table 1:** Characteristics of the study population (N=46).

Characteristics and categories			Participants, n (%)
**Gender**			
	Male	28 (61)	
	Female	18 (39)	
**Age (years)**			
	<30	13 (28)	
	30-55	31(68)	
	>55	2 (4)	
**BMI (kg/m^2^)**			
	Underweight (<18.5)	1 (2)	
	Normal (18.5-24.9)	20 (44)	
	Overweight (25-29.9)	5 (11)	
	Obese (>30)	6 (13)	
	Unknown (not reported)	14 (30)	
**Fagerström Test of Nicotine Dependence**			
	Very low (0-2)	18 (39)	
	Low (3-4)	18 (39)	
	Moderate (5-7)	10 (22)	
	High (8-10)	0 (0)	
**Measured average daily cigarette consumption**			
	Light (≤10 cigarettes per day)	31 (68)	
	Moderate (10-20 cigarettes per day)	13 (28)	
	Heavy (>20 cigarettes per day)	2 (4)	
**Self-reported average daily cigarette consumption**			
	Light (≤10 cigarettes per day)	24 (52)	
	Moderate (10-20 cigarettes per day)	20 (44)	
	Heavy (>20 cigarettes per day)	2 (4)	

### Characterization of Smoking Patterns

**Figure 3:**
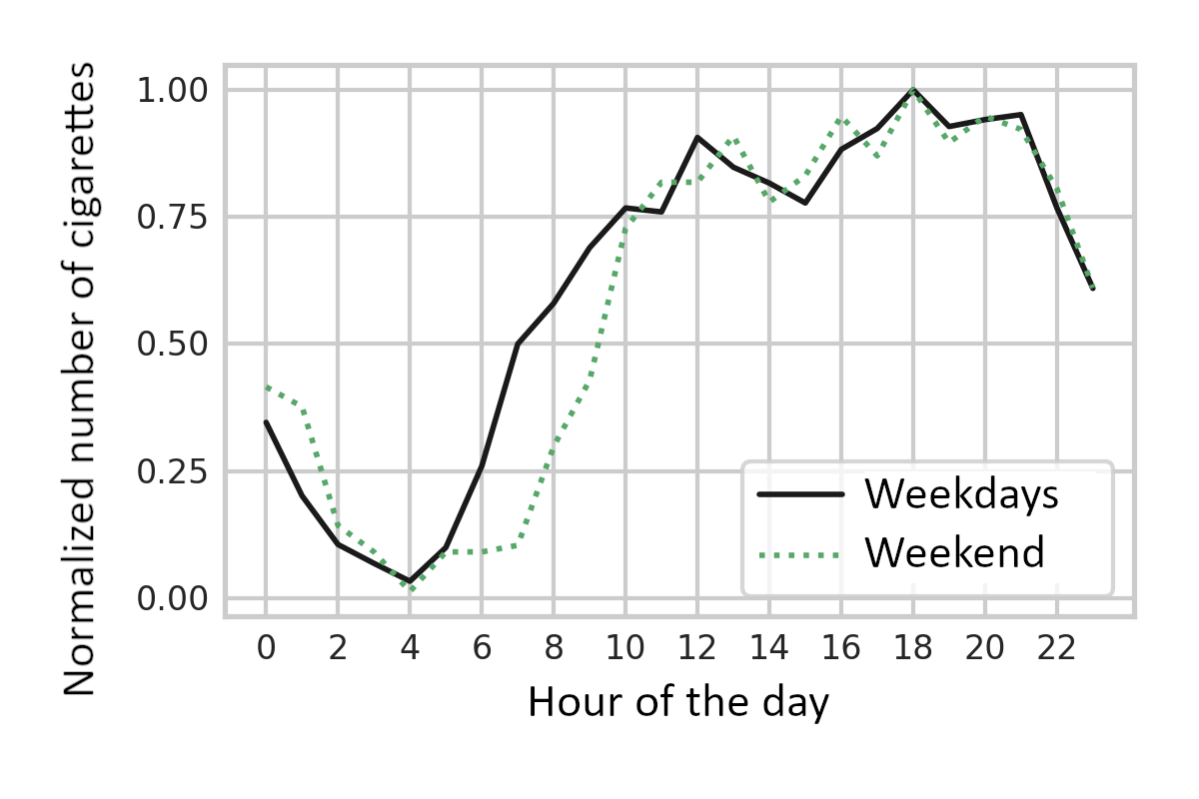
Average number of cigarettes smoked per hour of the day, maximum normalized, and split between weekdays and weekends.

Nation-wide surveys have shown differences in cigarette consumption between nonwork days and workdays [27]. As the smokers in this study were office workers, we assessed the differences between the days at work and at home, so the smoking records from all smokers during the study period of 4 weeks were aggregated and rescaled by the maximums for the weekdays and weekends. Figure 3 shows the distribution of cigarette consumption over 24 hours on weekdays and on weekends. The main finding is a 1- to 2-hour shift in the hourly cigarette consumption curves; on weekends, people smoke later, which is in line with a shift in sleeping times. For the weekdays, another peak is seen at 12 PM, which is usually the lunch time, and a valley at approximately 3 PM. For the weekends, however, 2 peaks in the afternoon are observed at around 1 PM and 4 PM, respectively. In addition, it can be observed from both curves that the number of cigarettes smoked generally increases later in the day, and most cigarettes are smoked around 6 PM.

### EMA Reports of Smoking

To complement the FTND score on the psychosocial dimensions of nicotine dependence, this study used smoking-contingent EMA surveys to assess 5 smoking-relevant feelings. According to the dimensional models, emotion consists of at least two distinct dimensions; that is, valence and arousal [28]. In the EMA survey, these 2 emotions were assessed on a 9-point rating scale (numerically from −4 to 4). Emotional valence describes the extent to which an emotion is positive or negative, whereas arousal refers to its intensity; that is, the strength of the associated emotional state ranging from extremely calm to extremely excited [29]. There were another 3 questions used to assess the strength of craving, enjoyment of cigarettes, and difficulty of resisting smoking, respectively, on a scale from 0 to 4. The lower the score, the less intense the feeling. In total, 1839 EMA annotations were logged into the database.

**Figure 4:**
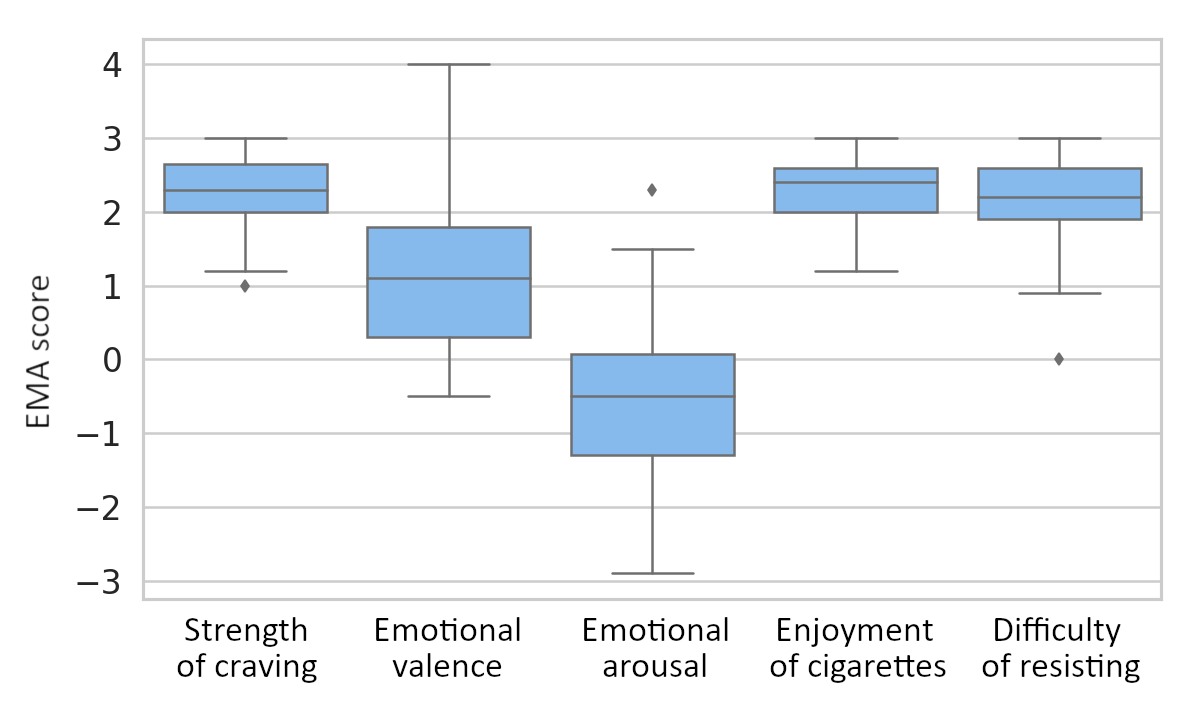
The distribution of self-assessed levels of 5 different ecological momentary assessment (EMA) questions related to smoking. Median is depicted as horizontal line inside each box, the box itself shows the IQR, and the whiskers end 1.5 times IQR away from the IQR. Outliers are depicted by black diamonds.

The answer distributions of the 5 EMA questions are shown in Figure 4. The distributions of craving, enjoyment, and difficulty of resisting smoking were very similar and were mostly >2, which is the middle of the scale. A Spearman correlation test was used to verify the associations among them. Table 2 lists the mean and SD, as well as the coefficients of correlation and significance level. Their craving for cigarettes was 2.4 (SD 0.7), their average enjoyment was 2.5 (SD 0.7), and the average difficulty of resisting smoking was 2.4 (SD 0.7). In addition, craving is strongly correlated with enjoyment with a tested coefficient of 0.73 and *P*<.001, and it is also correlated with the difficulty of resisting smoking. In addition, enjoyment and difficulty of resisting smoking are correlated with a coefficient of 0.51 with high confidence. This result shows that increasing craving levels for cigarettes results in more enjoyment of smoking and more difficulty in resisting cigarettes.

Regarding the 2 dimensions of emotion, smoking was generally reported to be associated with more positive feelings for office workers during their daily lives. The emotional valence was 1.6 (SD 1.8). Most of the time, smokers were in a nonexcited state with a mean value of −0.6, but similar to emotional valence, a large variation exists. More detailed distributions of EMA answer data can be found in Multimedia Appendix 1 Figures S1-S4. Emotional valence was also negatively correlated with emotional arousal (*P*<.001).

**Table 2:** Mean value, SD, and Spearman correlation coefficient, *P*, of 5 smoking-related feelings.

Parameters		Strength of craving	Emotional valence	Emotional arousal	Enjoyment of cigarettes	Difficulty of resisting
Value, mean (SD)		2.4 (0.7)	1.6 (1.8)	−0.6 (2)	2.5 (0.7)	2.4 (0.7)
**Correlation coefficients (*P* value)**						
	Strength of craving	N/A^a^	0.07	−0.08	0.73	0.65
	*P* value	N/A	.64	.61	<.001	<.001
	Emotional valence	N/A	N/A	−0.50	0.35	0.16
	*P* value	N/A	N/A	<.001	.02	.33
	Emotional arousal	N/A	N/A	N/A	−0.22	−0.00
	*P* value	N/A	N/A	N/A	.17	.99
	Enjoyment of cigarettes	N/A	N/A	N/A	N/A	0.51
	*P* value	N/A	N/A	N/A	N/A	<.001

^a^N/A: not applicable (duplication).

### Characterizing and Comparing the 2 Smoker Groups

Table 3 compares the characteristics of the 2 defined smoker type groups statistically. The 2 smoker groups were clustered based on objectively measured smoking rates, where moderate and heavy smokers were combined into 1 group (Table 1). Overall, 52% (16/31) of light smokers were men, whereas 80% (12/15) were men, in the moderate and heavy smoker groups. Although a lower percentage of male smokers were found in the light group, the difference was not significant (*P*=.10) when examined by the Fisher exact test. Regarding age, moderate and heavy smokers were 6 years older on average than light smokers in this study. In addition, the moderate and heavy groups tended to have longer smoking years, but this difference was not as significant as age. In addition, BMI and age at smoking initiation were not significantly different. Smokers, therefore, mostly initiate smoking in adolescence and are more likely to develop into moderate and heavy smokers as they smoke longer. Furthermore, the average FTND scores were significantly different between these 2 groups, with light smokers having 1.3 points lower mean scores. From the EMA answer comparison, it can be seen that craving, enjoyment, and difficulty in resisting smoking are all significantly stronger among moderate and heavy smokers than among light smokers. More positive and calm feelings were reported among moderate and heavy smokers.

**Table 3:** Statistics of the features across the 2 smoker groups. *P* values are calculated by the Mann-Whitney U test.

Type and feature (range)			Light smokers, mean (SD)		Moderate and heavy smokers, mean (SD)		*P* value
**Demographics**							
	Age (years)	33.7 (9.2)		39.6 (10.3)		.02	
	Smoking initiation age (years)	17.5 (4.0)		18.4 (4.2)		.23	
	Smoking years	16.2 (9.6)		21.2 (11.4)		.09	
	Fagerström Test of Nicotine Dependence (0 to 10)	2.7 (1.9)		4.0 (1.6)		.01	
**Ecological momentary assessment**							
	Strength of craving (0 to 4)	2.3 (0.8)		2.6 (0.7)		<.001	
	Emotional valence (−4 to 4)	1.2 (1.8)		1.8 (1.8)		<.001	
	Emotional arousal (−4 to 4)	−0.2 (1.9)		−1.0 (2.1)		<.001	
	Enjoyment of cigarettes (0 to 4)	2.3 (0.7)		2.6 (0.6)		.001	
	Difficulty of resisting smoking (0 to 4)	2.3 (0.7)		2.5 (0.7)		.002	

### Modeling the Count of Cigarettes in a 1-Hour Window

Cigarette smoking is a typical example of a recurrent event. The pattern of recurrent smoking events may depend on time-varying covariates. Meanwhile, demographics and background information such as age, gender, and nicotine dependence, which are time invariant over the time span of the experiment, also affect smoking patterns.

Modeling of the number of cigarettes in a 1-hour time window was performed with demographics (age, gender, and FTND) and timing of smoking (day of week and time of the day) as inputs. In total, there were 6654 such 1-hour windows during which 8631 cigarettes were smoked. The cigarette count in a 1-hour time window was affiliated timewise to the start of the time window. To decide on the selection of random effects, 3 Poisson mixed-effects models were compared with a baseline model, which assumes that the count of smoking events in a time window is constant (Table 4). When including the participants as the random intercept factor, the first mixed-effects model significantly improved with a *P* value <.001 in the analysis of variance test, which confirms that considerable between-participant variability exists in the data. Comparing the models where time of day (hour) was introduced as a random slope or fixed effect, resulted in hour being selected as a fixed factor because of its analysis of variance results and the smallest value for the Akaike information criterion. This model allows each participant to have a random intercept and has an hour of smoking as a fixed effect and was extended by age, gender, FTND, and day of the week as fixed factors.

Count of cigarettes = approximately hour + age + gender + Fagerström Test of Nicotine Dependence + day of week + (1|participant_ID) **(1)**

In Table 5, the coefficients and corresponding SEs of the fixed factors from the final modeling results are included together with their *P* values. From the results, we can see only hour of smoking (*P*=.02) and age (*P*=.005) turned out to be informative for the number of cigarettes smoked in a 1-hour time window. FTND, gender, and day of the week were not informative for repeated smoking behavior. The model was also extended by each EMA variable, but these variables did not significantly improve the model (Multimedia Appendix 1 Table S1).

**Table 4:** Analysis of variance test results of 3 Poisson mixed-effects models relative to a baseline model for the selection of the random intercept or slope effects.

Models	Akaike information criterion	*P* value (analysis of variance)
Count of cigarettes (approximately 1)	15853.0	N/A^a^
Count of cigarettes (approximately 1 + [1|participant_ID])	15754.1	<.001
Count of cigarettes (approximately 1 + [hour|participant_ID])	15754.5	.17
Count of cigarettes (approximately hour + [1|participant_ID])	15750.7	.02

^a^N/A: not applicable (reference model).

**Table 5:** Model coefficients, SEs on the coefficients, and *P* values for the dependent variables in the derived Poisson mixed-effects model.

Variable	Coefficient (SE)	*P* value
Intercept	−0.075 (0.11)	.48
Hour	0.005 (0.002)	.02
Age (years)	0.007 (0.003)	.005
Gender (male)	0.033 (0.052)	.53
Fagerström Test of Nicotine Dependence	−0.012 (0.015)	.43
Day of week	0.002 (0.006)	.67

## Discussion

### Principal Findings

The strength of this study is that it reports on smoking behavior by using an electric lighter that provides objective annotations of smoking and EMAs triggered by these events. This combination aims to provide more accurate annotations on both the timing and context of smoking when compared with retrospective self-reporting. This approach, when validated sufficiently, can potentially help smokers quit smoking by recommending interventions such as nicotine replacement therapy at the right time and context. The challenge, however, is to measure sufficient internal drivers and environmental factors related to smoking behavior to accurately model the known inter- and intravariability of smoking behavior. Ideally, models tailored to individuals must be developed, but these require extensive longitudinal data sets, which are not easy to obtain. This paper provides insights on smoking behavior with respect to timing, demographics, and relevantly timed EMAs and highlights potential variables and technologies for future studies.

In our data, we reported overestimation of self-reported smoking rates compared with measured smoking rates with the lighter. Reduced retrospective recall and lack of awareness of smoking behavior may have caused this [11,26]. However, our approach also relied on the compliance of participants to use the lighter for every smoking event. Therefore, the overestimation may be a result of suboptimal compliance. A recent study with this type of lighter showed that 92.2% of the smoking events were tracked by the lighter during 14 days of study among 22 participants [30]. They also found lower measured smoking rates and increased smoking rate variability compared with that in retrospective reporting. Therefore, we argue that using an electronic lighter provides a basis for annotation accuracy improvement.

The high variability of measured smoking rates within participants indicates the complexity of smoking behavior; for example, that habit is not the only driver of behavior. Similar variations in daily cigarette consumption were also reported by Hughes et al [25] using self-reported data. Time factors that we found to be important for modeling behavior are a delayed smoking pattern on the weekend and increasing smoking rates as the day progresses; that is, a sinusoidal pattern that has a maximum around dinner time. Of these 2, only hour of day was significant in the Poisson mixed-effects model, indicating that the found increasing smoking rates along the day are also characterized by increased repeated smoking in short time windows.

We compared the characteristics of light smokers and moderate to heavy smokers and found that nicotine dependence (FTND) and age were higher for heavier smokers but not for smoking initiation age and number of smoking years. Age was found to be a robust factor to describe smoking behavior, which was confirmed by the finding that it is the only significant static predictor remaining in the Poisson model. Gender on the other hand, did not show differences in smoking rate or smoking frequency. This is in line with the literature, although women were found to perceive more stress and nicotine withdrawal symptoms in a smoking cessation context, so gender may be important when modeling the risk of relapse [31,32].

Previous studies have suggested that people smoke cigarettes to regulate emotions and relieve negative emotions as reviewed by Kassel et al [33]. In our study, EMA annotations of craving, emotional valence, arousal, smoking enjoyment, and difficulty of resisting smoking were significantly different between light and moderate to heavy smokers but were not significant in the Poisson model to predict the number of cigarettes smoked in 1 hour. Higher craving has been linked to higher smoking incidence [34,35], and the difficulty of resisting smoking was found to be different among smoker types [36]. Emotional valence and arousal have been studied widely with respect to smoking behavior, and negative affect (NA) has been recognized as a nicotine withdrawal symptom and is correlated in some studies to increased smoking but is considered not a reliable antecedent of smoking, given that for example, stress influences NA as well [34]. Furthermore, NA and arousal seem to have a quadratic relationship with smoking probability, implying that linear models, such as the Poisson model in this paper, may perform suboptimally [37]. In addition, the effect of NA is diminished by other contextual factors such as other substance use including alcohol, which indicates that extensive experience sampling remains crucial [38]. The idea in this study was that every smoking event was annotated by an EMA. However, the ratio of EMA to smoking events was 21.29% (1839/8639), and more than half of the EMAs were not answered within a 1-hour window of smoking. This caused very few EMA-annotated smoking windows to be used in the Poisson models, resulting in low statistical power to find significance. A challenge is, therefore, to increase compliance and engagement in smokers when using EMAs, for example, with gamification.

To make effective smoking cessation tools, improved and extended data capture and modeling are needed. Our Poisson model predicted whether 1 to 4 cigarettes were smoked in a 1-hour window. For nicotine replacement therapy strategies, prediction models for risk of a single smoking event in time (no smoking vs smoking) may be equally relevant, but that requires EMA data also to be available for time windows without smoking, which was not the case in this study owing to its design. These risk of relapse models should be tailored to each smoker because we also found the *participant* to be a significant random effect in the Poisson model. Continuous sensing of human physiology with wearables has the potential for capturing nicotine withdrawal stress responses as precursor to smoking. However, the challenges of high intra- and interparticipant variability and privacy concerns should be tackled before wearables can be used as a validated tool [39]. EMA reactivity; that is, the phenomenon of triggering a smoking event by answering a smoking-related EMA, has been shown to be an issue in smoking cessation contexts [40]. In our study, the most of the EMAs were triggered by smoking and only in rare cases EMAs were triggered randomly. EMA reactivity was therefore considered not an issue and was treated as another smoking cue modulated by the studied factors. However, when the focus shifts toward cessation tools, finding proxies for states derived from EMA that can be measured continuously and nonintrusively may become important. Examples of these are mobile health measures such as smartphone use and web-based activity that have the potential as a proxy for mood state. Like many other studies, this study may suffer from selection bias toward motivated and tech-savvy participants. Future model development and validation should be performed in larger trials, in which smoker population characteristics are matched. The resulting increased variety of smokers would also facilitate to learn which subpopulations benefit most from the current modeling approach.

### Conclusions

This study reported on the high potential and challenges of using an electronic lighter for smoking annotation and smoking-triggered EMAs in an ambulant environment. It is expected that to develop effective intervention strategies for smoking cessation, research needs to shift from population-based data sets based on self-reporting to richer data sets with objective environmental, physiological, and behavioral sensing so that individualized prediction models for relapse can be developed. We contributed to this by characterizing smoker types and by modeling smoking frequency using demographic, timing, and EMA data.

## Appendix

Multimedia Appendix 1 Supplementary materials.

## Abbreviations

CPD: cigarette per day
EDiT: Enabling Digital Transformation
EMA: ecological momentary assessment
FTND: Fagerström Test of Nicotine Dependence
NA: negative affect
