# Supplementary material for: Characterizing and Modeling Smoking Behavior Using Automatic Smoking Event Detection and Mobile Surveys in Naturalistic Environments: Observational Study
Source: JMIR Mhealth Uhealth. 2022 Feb 18;10(2):e28159. doi: 10.2196/28159 (PMC8900898; doi:10.2196/28159)
Supplement: Multimedia Appendix 1 [file mhealth_v10i2e28159_app1.docx]

# Supplementary materials


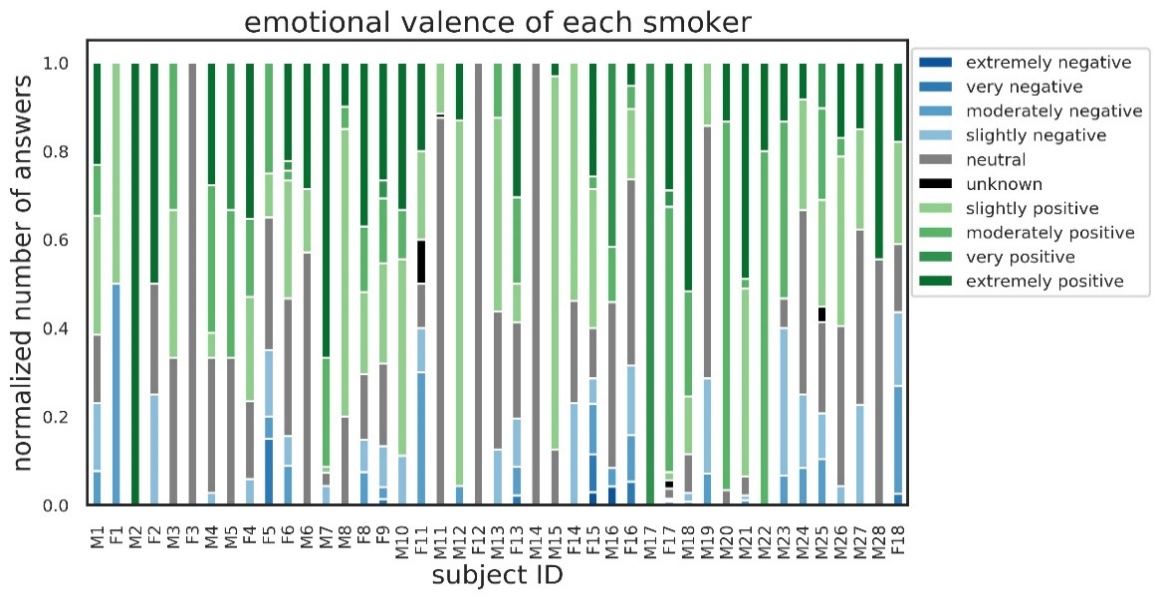


Figure S1. The distributions of self-reported emotional valence for each smoker.


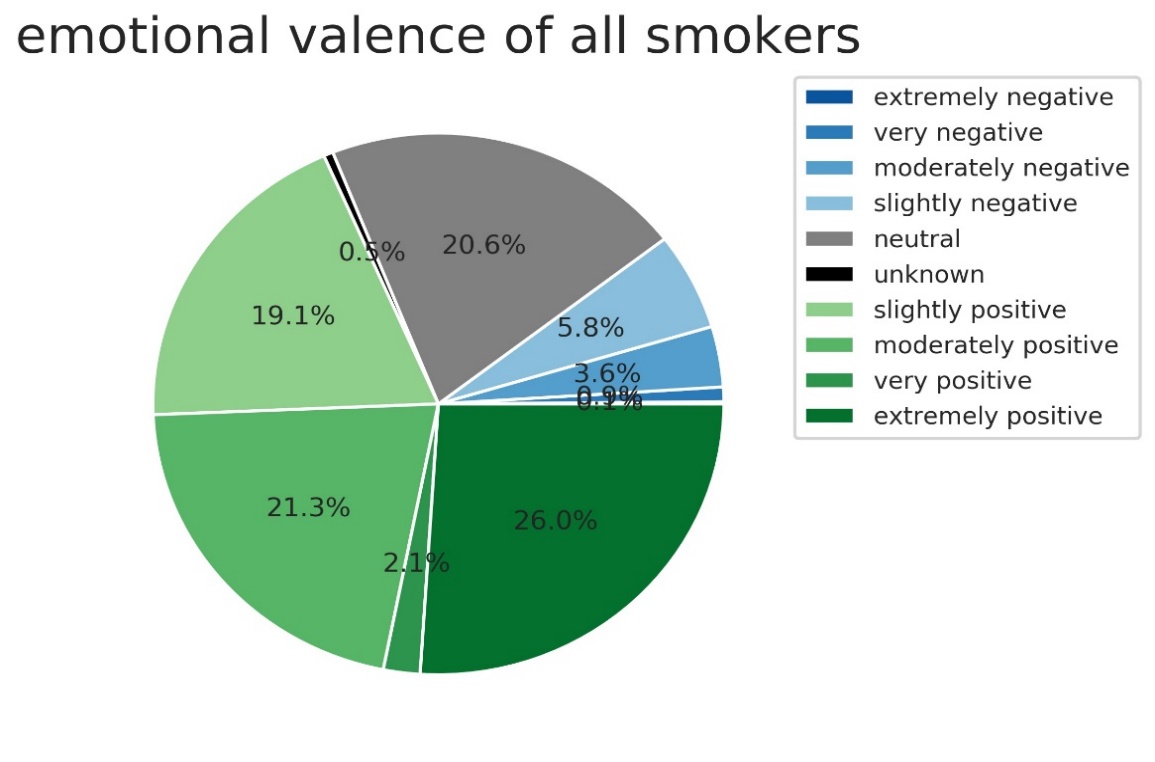


Figure S2. The distribution of self-reported emotional valence for all smokers combined.


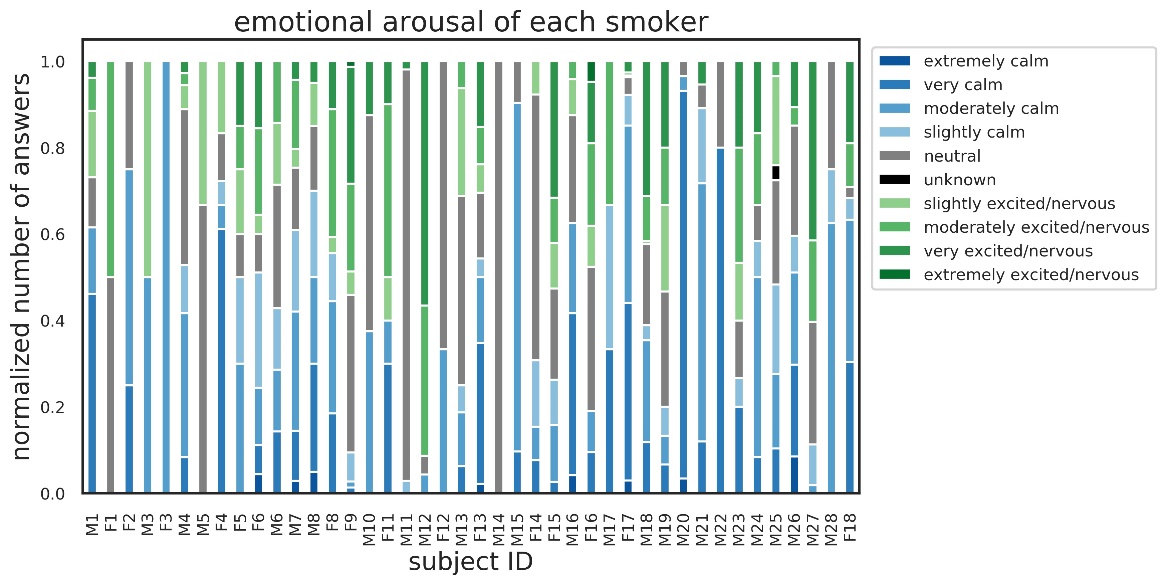


Figure S3. The distribution of self-reported emotional arousal for each smoker.


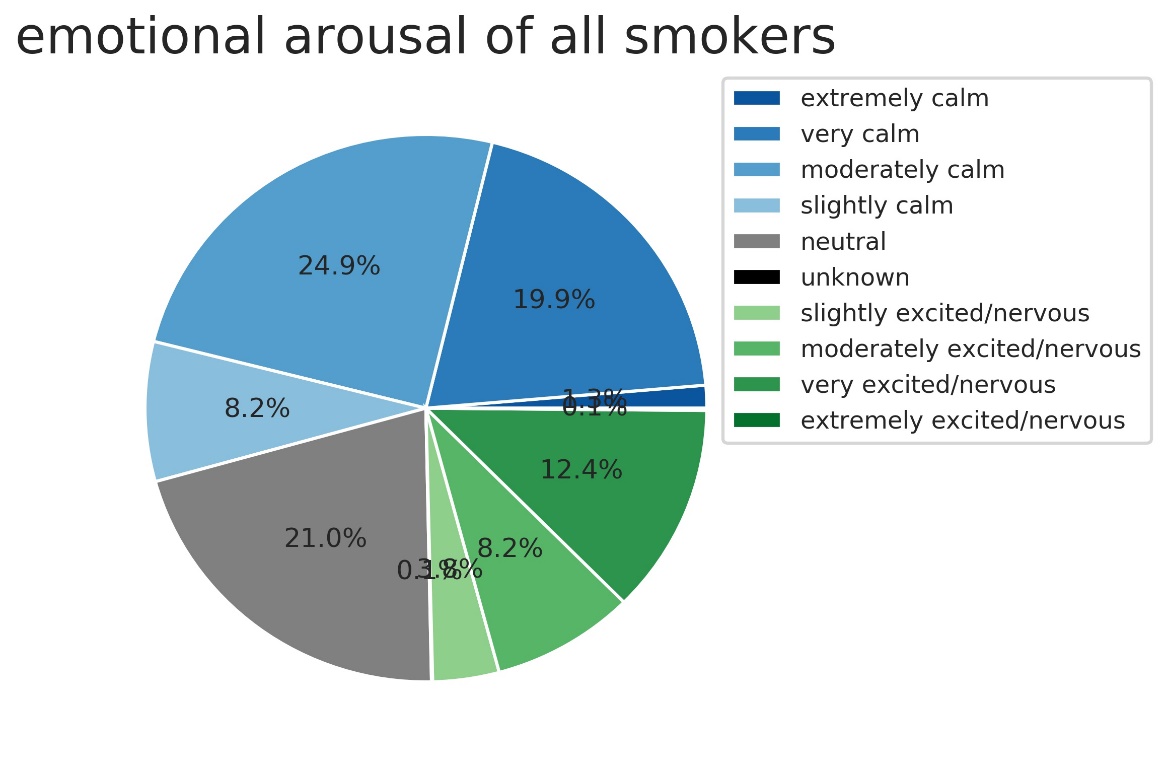


Figure S4. The distribution of self-reported emotional arousal for all smokers combined.

Table S1. Comparing 5 Poisson mixed effects models using addition of EMA variables against the baseline model.

| **Model** | **AIC** | ***P* (ANOVA)** | **Coefficient ± S.E**  **(Model)** | **P (Model)** |  |
| --- | --- | --- | --- | --- | --- |
| count_of_cigarettes ~ age + hour + +(1\|subject_id) | 679.72 |  |  |  | |
| + Strength of craving | 681.56 | .69 | -0.035±0.076 | .65 | |
| + Emotional valence | 681.59 | .72 | -0.009±0.028 | .73 | |
| + Emotional arousal | 681.35 | .96 | 0.007±0.003 | .99 | |
| + Enjoyment of cigarettes | 681.42 | .55 | 0.033±0.052 | .55 | |
| + Difficulty of resisting smoking | 681.42 | .59 | -0.012±0.015 | .57 | |
